# WORKFORCE ENHANCEMENTS FOR HEALTHY AGING AND INDEPENDENT LIVING: COLLABORATION TO IMPROVE HEALTH CARE

**DOI:** 10.1093/geroni/igae098.0065

**Published:** 2024-12-31

**Authors:** Janice Knebl, Jennifer Severance, Sara Murphy

**Affiliations:** University of North Texas Health Science Center, Fort Worth, Texas, United States; University of North Texas Health Science Center, Fort Worth, Texas, United States; University of North Texas Health Science Center, Fort Worth, Texas, United States

## Abstract

There are 48 Geriatric Workforce Enhancement Programs (GWEP) funded by the Health Resources and Services Administration in 2019 to integrate geriatrics education into primary care training, increase patient and family engagement, and improve health outcomes. The University of North Texas Health Science Center (UNTHSC) GWEP collaborates with a county hospital, a private university, an Area Agency on Aging, and other community-based organizations to align education and training enhancements and activities with community needs. Using a multipronged strategy focused on engaging local primary care and long-term care teams to identify community needs and learner preferences, a GWEP team of interprofessional faculty and community and health care partners combined critical resources to develop and enhance curricula in geriatric best practices using the Age-Friendly Health Systems 4Ms framework. Partners also developed an infrastructure to reach rural and underserved communities using learning technologies, social media platforms, and community health educators. This presentation will address the impact on medical professions students and professionals, the community partnerships that were built, and the advancement of interprofessional geriatric education.

